# A four-gene prognostic signature for predicting the overall survival of patients with lung adenocarcinoma

**DOI:** 10.7717/peerj.11911

**Published:** 2021-09-23

**Authors:** Lei Liu, Huayu He, Yue Peng, Zhenlin Yang, Shugeng Gao

**Affiliations:** Department of Thoracic Surgery, National Cancer Center/National Clinical Research Center for Cancer/ Cancer Hospital, Chinese Academy of Medical Sciences and Peking Union Medical College, Beijing, China

**Keywords:** Lung adenocarcinoma, Prognostic model, Risk score, Overall survival, TCGA, GEO

## Abstract

**Background:**

The prognosis of patients for lung adenocarcinoma (LUAD) is known to vary widely; the 5-year overall survival rate is just 63% even for the pathological IA stage. Thus, in order to identify high-risk patients and facilitate clinical decision making, it is vital that we identify new prognostic markers that can be used alongside TNM staging to facilitate risk stratification.

**Methods:**

We used mRNA expression from The Cancer Genome Atlas (TCGA) cohort to identify a prognostic gene signature and combined this with clinical data to develop a predictive model for the prognosis of patients for lung adenocarcinoma. Kaplan-Meier curves, Lasso regression, and Cox regression, were used to identify specific prognostic genes. The model was assessed via the area under the receiver operating characteristic curve (AUC-ROC) and validated in an independent dataset (GSE50081) from the Gene Expression Omnibus (GEO).

**Results:**

Our analyses identified a four-gene prognostic signature (*CENPH*, *MYLIP*, *PITX3*, and *TRAF3IP3)* that was associated with the overall survival of patients with T1-4N0-2M0 in the TCGA dataset. Multivariate regression suggested that the total risk score for the four genes represented an independent prognostic factor for the TCGA and GEO cohorts; the hazard ratio (HR) (high risk group *vs* low risk group) were 2.34 (*p* < 0.001) and 2.10 (*p* = 0.017). Immune infiltration estimations, as determined by an online tool (TIMER2.0) showed that CD4+ T cells were in relative abundance in the high risk group compared to the low risk group in both of the two cohorts (both *p* < 0.001). We established a composite prognostic model for predicting OS, combined with risk-grouping and clinical factors. The AUCs for 1-, 3-, 5- year OS in the training set were 0.750, 0.737, and 0.719; and were 0.645, 0.766, and 0.725 in the validation set. The calibration curves showed a good match between the predicted probabilities and the actual probabilities.

**Conclusions:**

We identified a four-gene predictive signature which represents an independent prognostic factor and can be used to identify high-risk patients from different TNM stages of LUAD. A new prognostic model that combines a prognostic gene signature with clinical features exhibited better discriminatory ability for OS than traditional TNM staging.

## Introduction

Lung cancer has the highest incidence and mortality rates of all forms of cancers in China. Furthermore, it was estimated that in 2015, there were 787,000 new cases and 630,500 deaths of lung cancer in China (Gao et al., 2020). Data from the United States of America showed that in 2019, 228,150 patients were newly diagnosed with lung cancer while 142,670 patients died from this condition; furthermore, only 25% of patients with non-small cell lung cancer (NSCLC) survived longer than 5 years (Ettinger et al., 2019). There are two major subtypes of NSCLC: lung adenocarcinoma (LUAD) and lung squamous carcinoma; the former has now surpassed the latter as the most common pathological subtype among men in certain Asian populations (Chinese, Japanese) and in North America (USA, Canada). In women, however, adenocarcinoma is the dominant histological type almost everywhere, except for Poland and England (Travis et al., 2004). The prognosis of LUAD is related to a variety of factors, such as the TNM (tumor, regional lymph node, metastasis) stage (as used in the present study), tumor differentiation, and pathological subtype; these factors are widely used to guide clinical decision making. However, these factors are not sufficient to accurately evaluate the prognosis of patients with this disease. The 5-year overall survival (OS) of patients with IA stage LUAD was reported to be just 63% (Oskarsdottir et al., 2016), Consequently, there is a critical need to identify new biomarkers with which to evaluate prognostic outcome.

Gene sequencing has developed rapidly over the last few years and has allowed us to identify an increasing number of molecular prognostic factors for several cancers, including colon cancer, lung cancer, and breast cancer (Pei et al., 2020; Yang et al., 2020; Zhang et al., 2020a). Indeed, recent studies have demonstrated that genomic data are superior to the traditional staging system for estimating the risk of a worse prognosis and predicting the benefit of adjuvant chemotherapy (He & Zuo, 2019).

**Figure 1:**
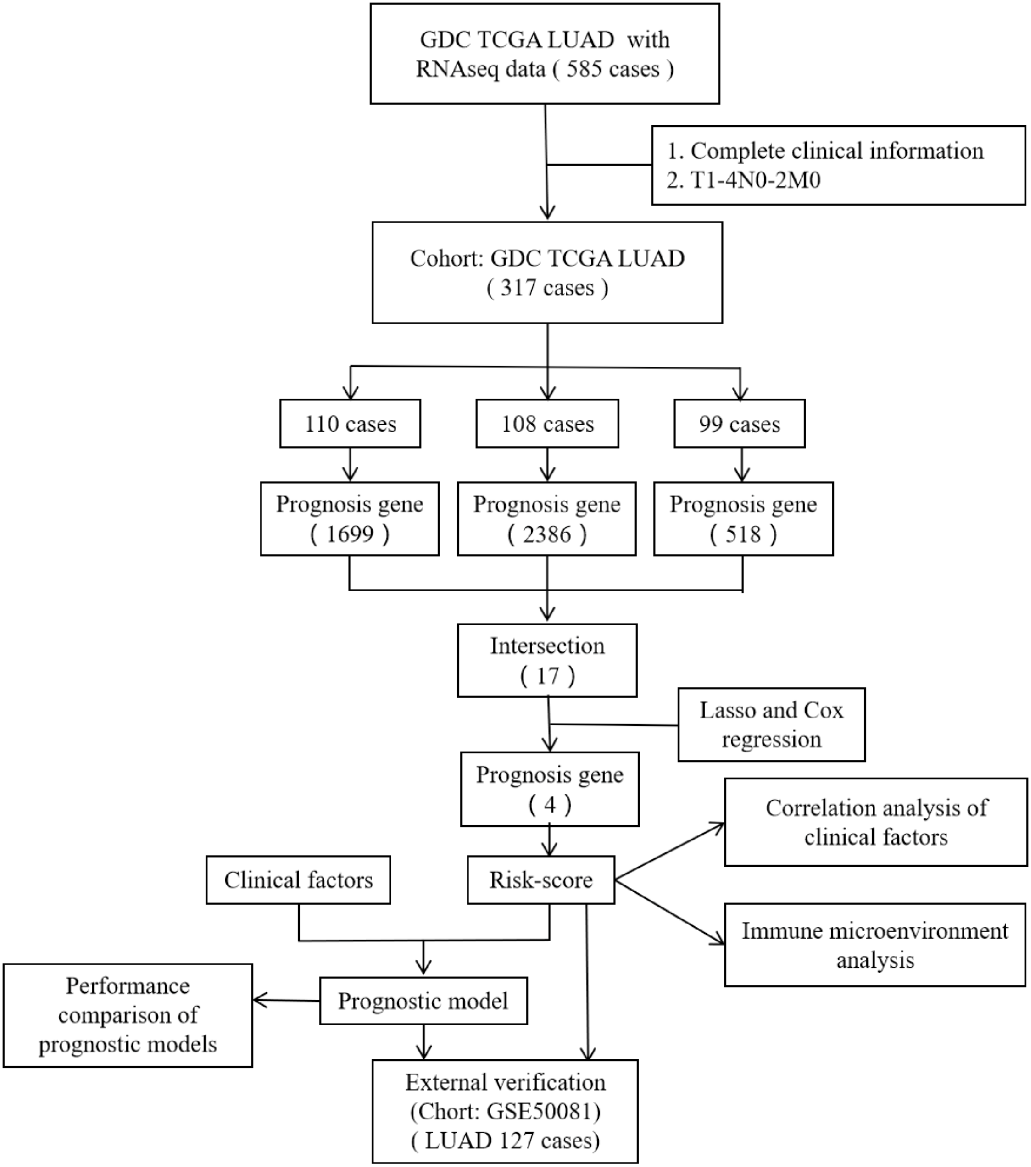
The flow diagram of this study. A total of 317 patients were included in the training set and 127 patients were included in the validation set.

However, most researchers screen prognosis-related genes from differentially expressed gene sets or only perform one round of screening (He & Zuo, 2019; Song et al., 2021; Zuo et al., 2019). We believe that such screening methods may miss certain genes that are not differentially expressed but are important for prognosis. In the present study, we performed prognostic gene screening directly from specific gene profiles and used a random grouping method that was intended to identify a more reliable set of prognosis-related genes. Four genes (*CENPH*, *MYLIP*, *PITX3*, and *TRAF3IP3)* were eventually identified; these were associated with tumor-infiltrating immune cells, the proliferation of tumor cells, and cell adhesion. Risk-scores were calculated using the four gene mRNA expression data and represented an independent prognostic factor for LUAD. This allowed us to develop a prognostic model which was then validated in an independent cohort of patients. Figure 1 shows the flow diagram of this study.

## Materials & Methods

### Data sources

We downloaded gene expression data (FPKM format), clinical information, and survival data, of patients with LUAD from The Cancer Genome Atlas (TCGA) from the University of California Santa Cruz Xenabrowser (UCSC Xena, https://xenabrowser.net/datapages/P) (Goldman et al., 2020) to act as a training cohort. We also acquired gene expression data and corresponding clinical information for the validation cohort under accession number GSE50081 from the Gene Expression Omnibus (GEO, http://www.ncbi.nlm.nih.gov/geo/). This validation dataset included 127 cases of lung adenocarcinoma.

Patients were included in our analyses if (1) clinical information (gender, age, T-stage, N-stage, M-stage), survival data (follow-up time and survival status), and gene expression information (mRNA expression levels) were complete and (2) the TNM stage was T1-4N0-2M0. We excluded cases if the histological type was not LUAD. After screening, a total of 317 patients were included in the training set and 127 patients were included in the validation set.

### Prognostic gene signature screening

The set of training samples were randomly divided into three groups. The levels of gene expression in each group were then divided into high and low expression groups using the median value as the cutoff point. Next, we used a univariate Cox proportional hazard regression model to determine the association between gene expression and OS in each group; genes with a *p*-value < 0.05 (following the log rank test) were defined as being prognosis-related. We then identified the intersection between the three groups to identify candidate genes. Lasso regression and a multivariate Cox regression model were both used to carry out further screening; gene expression was used as continuous variable parameter. This additional analysis identified a prognostic gene signature consisting of four genes: *CENPH*, *MYLIP*, *PITX3*, and *TRAF3IP3*.

In the training set, the gene expression levels (z-score) of these four genes were used as covariates in a multivariate Cox regression model were applied. We then calculated risk scores based on gene expression levels and risk coefficients. Then, the entire cohort was divided into a high-risk group and a low-risk group using the median value as the threshold. Next, we compared survival data, clinical characteristics, and the immune microenvironment between the two different risk-groups and then validated the model in an external dataset. The immune microenvironment was estimated by TIMER2.0 (http://timer.comp-genomics.org/) (Li et al., 2020b), a public database, and six immune infiltration lymphocyte estimations were obtained.

### Development and validation of the prognostic model

A multivariate Cox regression model was used to develop the prognostic mode. External validation was performed in an independent dataset. The degree of differentiation exhibited by the models was compared by comparing the area under the receiver operating characteristic curve (AUC-ROC). Calibration was evaluated using calibration curves.

### Statistical analysis

All statistical analyses were performed with R software version 4.0.3. OS data were calculated using Kaplan–Meier curves, and the statistical difference between different groups was determined by the log-rank test. The influence of different parameters on OS was evaluated by univariate and multivariate Cox proportional hazard regression models. Hazard ratios (HRs) and the 95% confidence intervals (CIs) were generated using Cox proportional hazards models. In addition, receiver operating characteristic (ROC) analysis was carried out to compare the predictive accuracy of models. Pearson’s Chi-squared test was performed to compared the population demographics. A *P*-value < 0.05 was determined to be statistically significant.

## Results

### Demographics of the study population

According to the inclusion and exclusion criteria, 317 cases were included in the TCGA cohort, while 127 cases were included in GSE50081. The median age of the two cohorts were 65-years and 70-years, respectively. The Stage of 5 patients in the TCGA cohort were not recorded; we defined these according to the 7th edition of the Union for International Cancer Control (UICC) TNM stage. The frequencies of T stage (*p* = 0.002), N stage (*p* < 0.001), and Stage (*p* < 0.001) in the two cohorts were different. Patient demographics are shown in Table 1.

**Table 1:** The patient demographics. T stage, N stage, Stage (TNM stage) are according the 7th or 6th UICC TNM stage.

**Variables**		**TCGA**	**GSE50081**	**X** ^**2**^	***P*-value**
**All**		317	127		
**Age (years)**					
	Median	66 (33–87)	70 (40–86)		
**Gender**					
	Male	154 (48.6%)	65 (51.2%)	0.15	0.696
	Female	163 (51.4%)	62 (48.8%)		
**T stage**					
	T1	98 (30.9%)	43 (48.6%)	12.48	0.002
	T2	180 (56.8%)	82 (33.9%)		
	T3+4	39 (12.3%)	2 (1.6%)		
**N stage**				21.13	<0.001
	N0	203 (64.0%)	94 (74.0%)		
	N1	67 (21.1%)	33 (26.0%)		
	N2	47 (14.8%)	0		
**Stage**				28.69	<0.001
	I	172 (54.3%)	92 (72.4%)		
	II	86 (27.1%)	35 (27.6%)		
	III	59 (18.6%)	0		

### Identification of a four-gene prognostic signature

**Figure 2:**
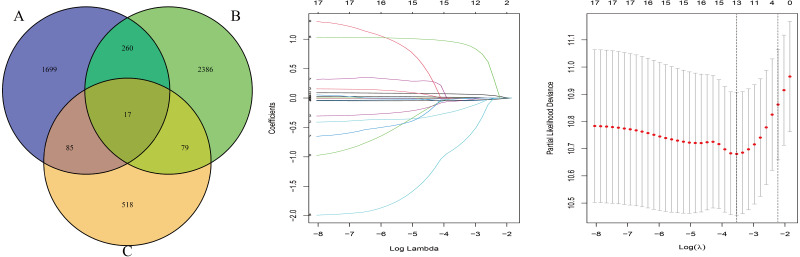
The gene screening process. The Venn diagram shows the intersection of the number of genes associated with prognosis in three randomized groups (A, B, C). The other two were the visualization of Lasso regression.

**Figure 3:**
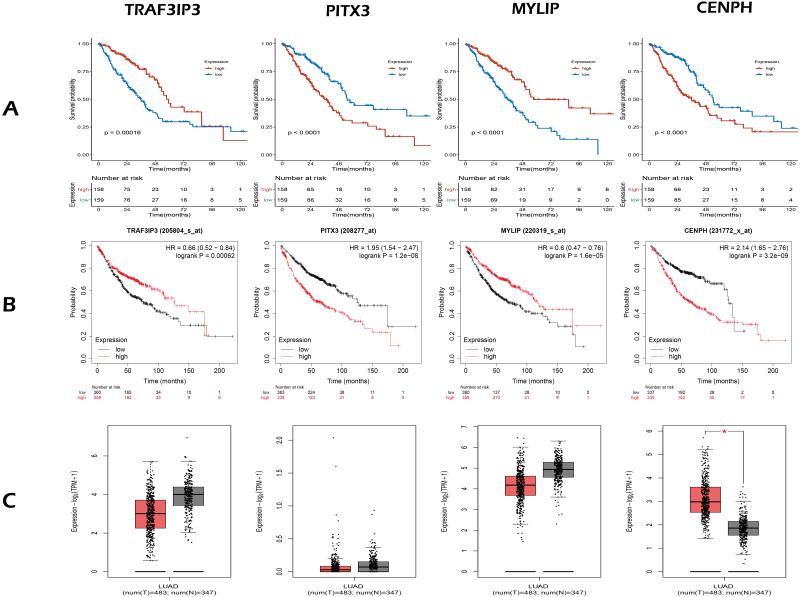
The expression and survival analysis of four gene. (A) The Kaplan–Meier curves for four genes in TCGA cohort of present study. (B) The Kaplan–Meier curves for four genes using online analysis site Kaplan–Meier Plotter. (C) The comparison of the expression of four genes between lung adenocarcinoma and normal tissue, using online analysis site GEPIA2; the red box is tumor, the gray box is normal tissue. In survival analysis (A, B), median of the expression of each gene was the cut-off.

The OS-related genes were identified from the training set using the Cox log-rank test and cut-off threshold of *P* < 0.05. The three randomized groups (A, B, C) contained 2061, 2742, and 699 OS-related genes, respectively. By identifying the intersection between the three gene sets, we identified 17 genes as candidate prognostic genes. Additional analysis, using Lasso and Cox stepwise regression, identified four prognostic genes: including two high risk genes (HR > 1; *CENPH* and *PITX3)* and two protective genes (HR < 1; *MYLIP* and *TRAF3IP3*). Figure 2 shows the gene screening process. Further details of the Cox regression model used to identify the four-genes are shown in Table 2. The Kaplan–Meier curves for the four genes in TCGA are shown in Fig. 3A.

**Table 2:** The results of multivariate cox regression analysis for the four gene expressions. The details of multivariate Cox regression model, the expression of four genes were used as continuous variables.

**GENE**	**Coefficient**	**HR**	**95% CI**	***P*-value**	**Prognosis**
***TRAF3IP3***	−0.28593	0.7513	0.5943∼0.9498	0.016833	Protective
***PITX3***	0.21193	1.2361	1.0961∼1.3939	0.000547	Risky
***MYLIP***	−0.47968	0.6190	0.4892∼0.7831	6.42e−05	Protective
***CENPH***	0.26692	1.3059	1.1248∼1.5162	0.000458	Risky

Next, we validated the effect of the four prognostic genes on OS using the Kaplan–Meier Plotter (https://kmplot.com/analysis/) and obtained the same conclusion, as shown in Fig. 3B. Online analysis of the Gene Expression Profiling Interactive Analysis (GEPIA2, http://gepia2.cancer-pku.cn/) public database (Tang et al., 2019) showed that the expression of *CENPH* was up-regulated in tumors when compared to normal tissue and that expression levels increased with increasing tumor stage. In contrast, the other three genes were down-regulated but without statistical significance (—Log2FC— Cutoff = 1, *p*-value Cutoff = 0.01), as shown in Fig. 3C.

### The risk score for the four-gene signature predicted the OS of patients with LUAD

**Figure 4:**
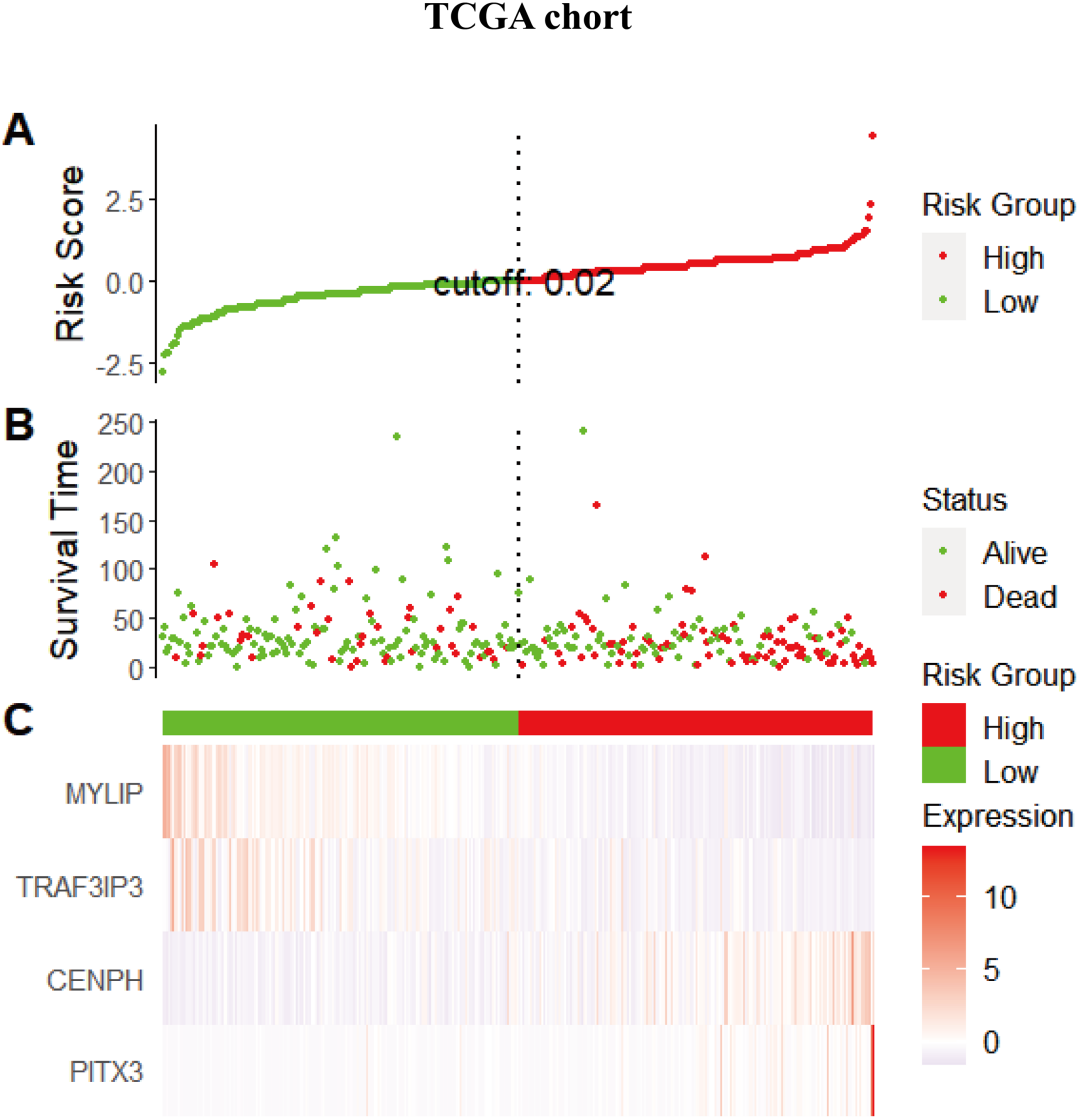
The distribution of the risk scores, gene expression levels, and the survival status of patients in the training set. (A) All samples sorted according to the risk score from low to high, and divided into high-risk group and low-risk group with the median value of 0.02 as the cutoff value. (B) The survival status and time of the high and low risk groups. (C) The expression levels of four genes in the high - and low-risk groups.

Considering that the expression levels of the four identified genes exhibited a correlation with prognosis, we next explored the combined prognostic effect of these genes by using the ggrisk package in the R environment. Each patient was given an individual risk score by using a prognostic Cox regression model according to the expression level and its corresponding coefficients in the training set. Patients were then divided into a high risk group and a low risk group using the median risk score (0.02066664) as a cutoff. The same risk score method was performed for the validation set but using the median risk score (−0.00797257) as the cutoff point. Figure 4 shows the distribution of the risk scores, gene expression levels, and the survival status of patients in the training set. Two protective genes (*MYLIP*, *TRAF3IP3*) were highly expressed in the low-risk group, while risky genes (*CENPH, PITX3*) were highly expressed in the high-risk group.

Equations (1) and (2) show the formulae for calculating the risk score for the training and validation sets, respectively. These formulae used the same coefficients; these originated from the Cox model for gene risk as fitted to the training set (Table 2).

(1) \begin{eqnarray*}\text{Risk score}~(\text{Training set})=TRAF3IP3\ast (-0.28593)+PITX3\ast (0.21193)\nonumber\\\displaystyle \hspace*{96.0pt}+MYLIP\ast (-0.47968)+CENPH\ast (0.26692)\end{eqnarray*}

(2) \begin{eqnarray*}\text{Risk score}~(\text{validation set})=TRAF3IP3\ast (-0.28593)+PITX3\ast (0.21193)\nonumber\\\displaystyle \hspace*{96.0pt}+MYLIP\ast (-0.47968)+CENPH\ast (0.26692)\end{eqnarray*}

**Figure 5:**
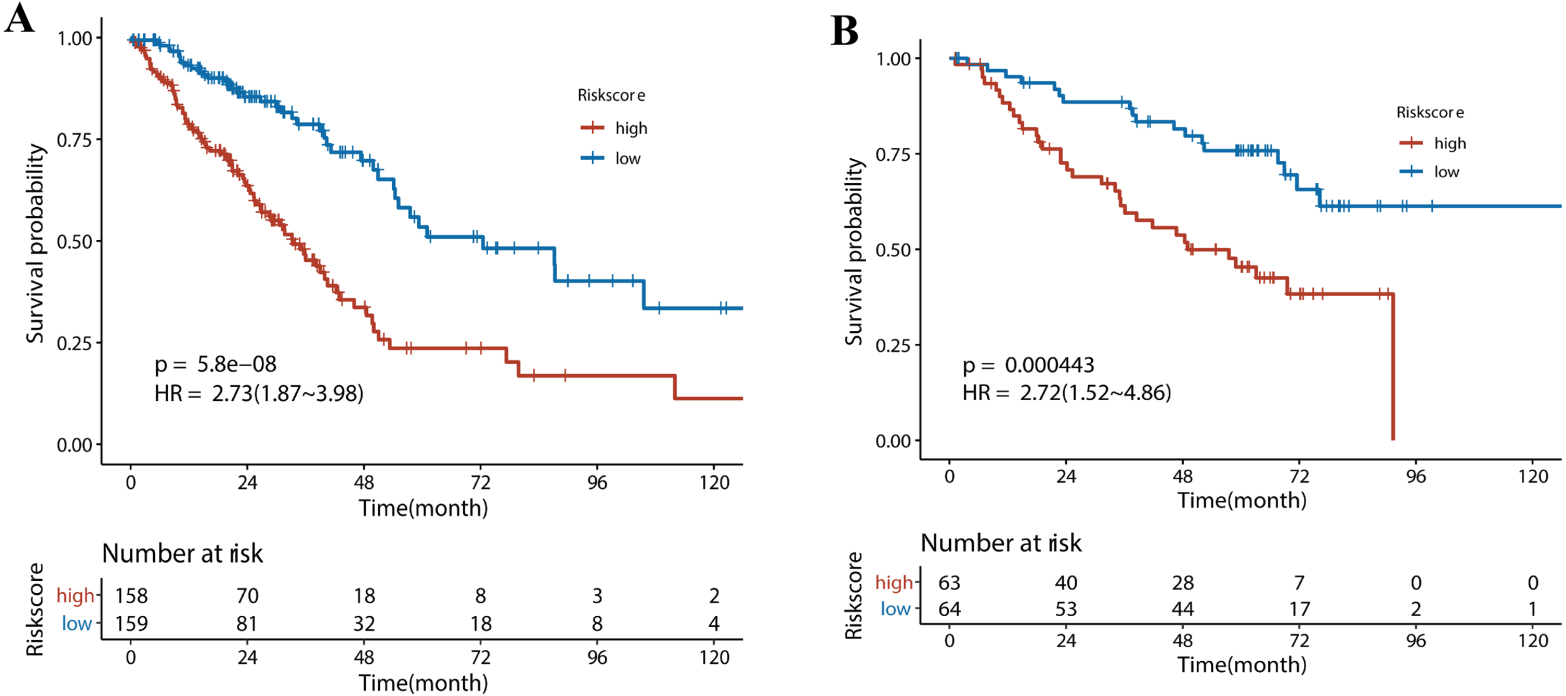
The survival analysis for different risk group in training set and validation set. (A) Kaplan–Meier survival analysis of risk score in TCGA cohort. (B) Kaplan–Meier survival analysis of risk score in the GSE50081 cohort. Patients in the high-risk group had a significantly shorter OS than those in the low-risk group in both cohorts.

**Figure 6:**
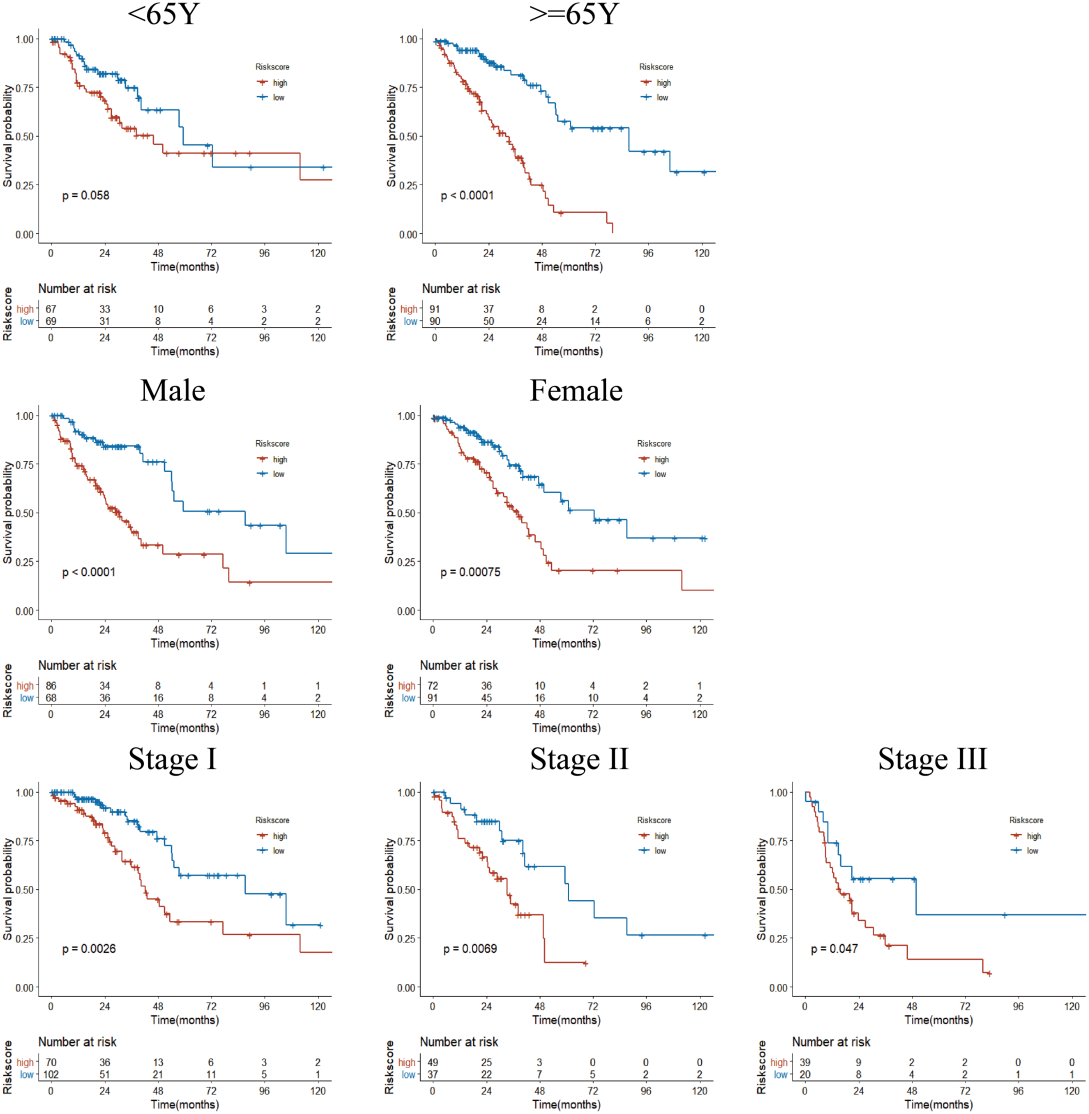
Subgroup Kaplan–Meier survival analysis in TCGA cohort. Stage represents TNM stage. The high-risk group had a shorter survival time than the low-risk group in almost every subgroup, although the *p*-value did not reach statistical significance in patients younger than 65 years.

**Figure 7:**
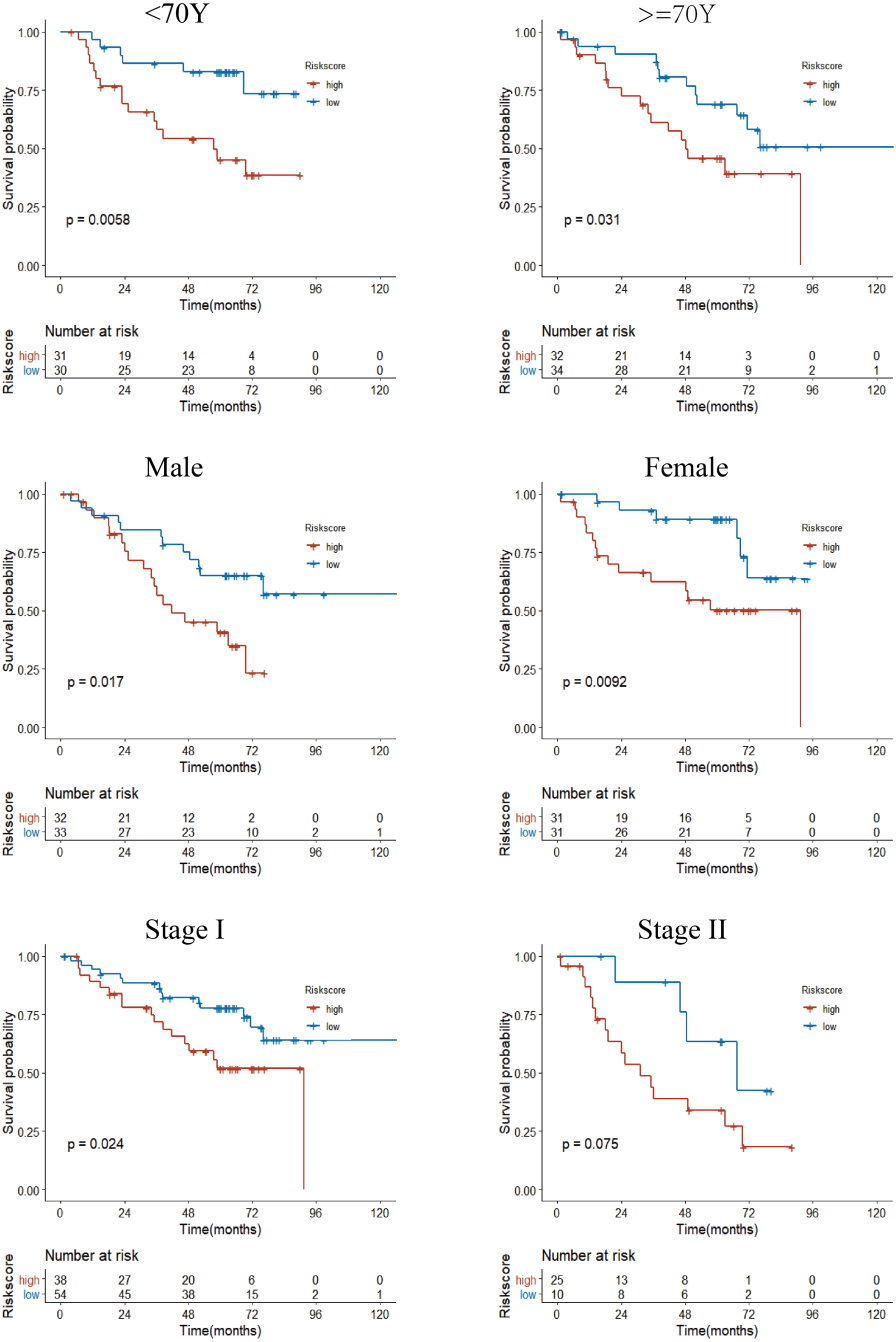
Subgroup Kaplan–Meier survival analysis in the GSE50081 cohort. Stage represents TNM stage. The high-risk group had a shorter survival time than the low-risk group in almost every subgroup, although the *p*-value did not reach statistical significance in patients with Stage II.

Next, we performed univariate Cox regression analysis. Kaplan–Meier curves (Fig. 5) showed that patients in the high-risk group had a significantly shorter OS than those in the low-risk group (Training set: HR = 2.73, 95% CI [1.87–3.98], *P* < 0.001; Test set: HR = 2.72, 95% CI [1.52∼−4.86], *P* <  0.001). Subgroup analysis were further performed. In the training set (Fig. 6), the high-risk group had a shorter survival time than the low-risk group in almost every subgroup (Stage I, *p* = 0.003; Stage II, *p* = 0.007; Stage III, *p* = 0.047), although the *p*-value did not reach statistical significance in patients younger than 65 years (*p* = 0.058). Except for the Stage II subgroup (*p* = 0.075), all other subgroups showed significant survival differences in the test set (Fig. 7).

### Risk-score was an independent prognostic factor for OS

**Figure 8:**
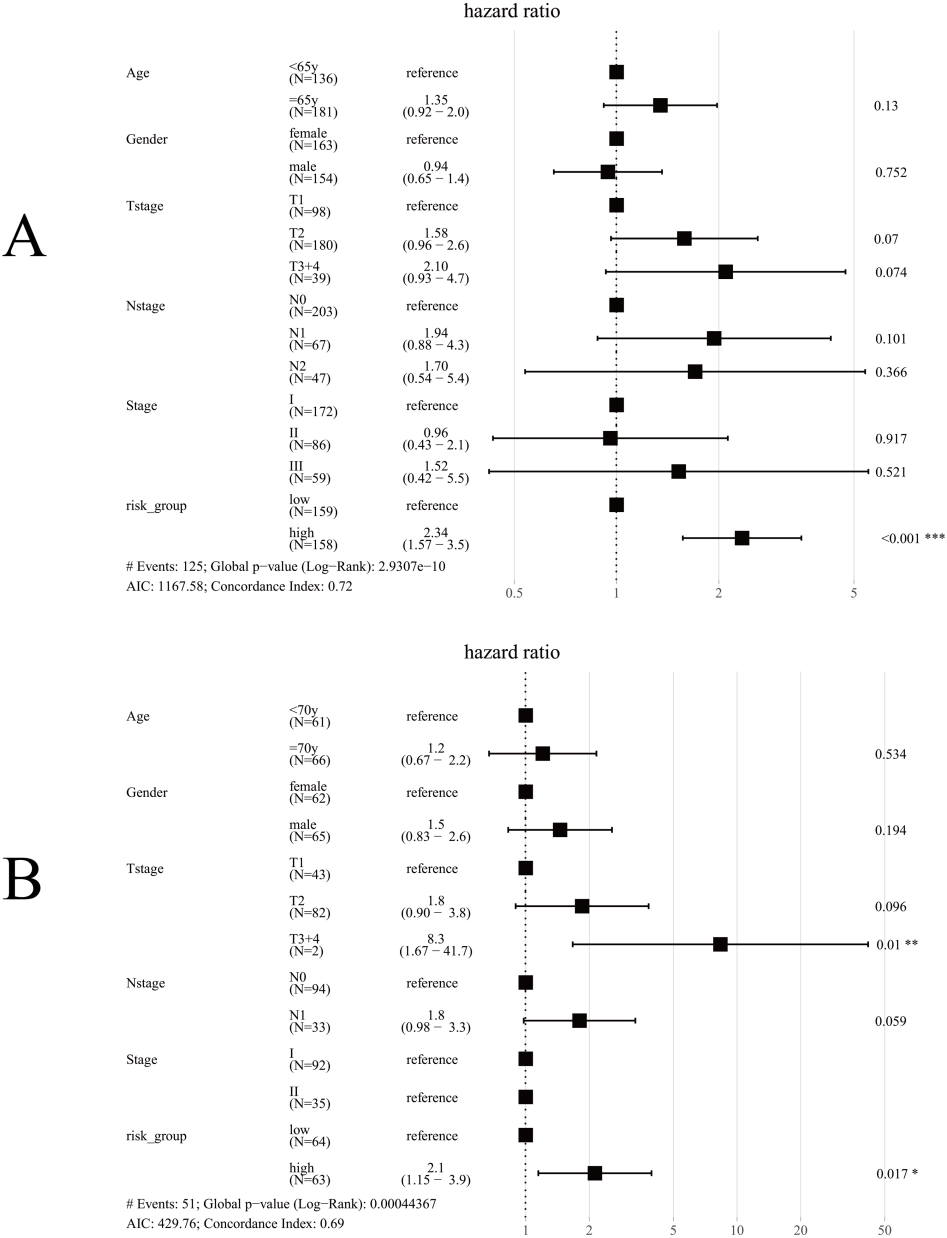
Forest plot for multivariate Cox regression. (A) In TCGA cohort, only risk-group was an independent prognostic factor; (B) In the GSE50081 cohort, risk-group and T3+4 were independent prognostic factors; Note: Stage and N-stage in the GSE50081 cohort almost coincided exactly, patients with Stage I were all N0, and 35 patients were Stage II, only 2 samples were N0, consequently, multivariate analysis did not obtained result for Stage.

Univariate Cox regression analysis for TCGA cohort suggested T stage (*p* < 0.001), N stage (*p* < 0.001), Stage (*p* < 0.001) and Risk group (*p* < 0.001) were significantly associated with OS (Table 3). The same results were obtained in the GES50081 cohort (Table 4). Next, we used the risk-group and other clinical factors (including age, gender, T-stage, N-stage and Stage) as covariates and created a multivariate Cox regression model for the training set and the validation set (Fig. 8). We found that only the risk-group was an independent prognostic factor in the training set, while risk-group and T3+4 were independent prognostic factors in the validation set. Stage and N stage in the validation set almost coincided exactly. Patients with Stage I were all N0, and 35 patients were Stage II; only 2 samples were N0. Consequently, multivariate analysis for Stage did not obtained result for Stage. After adjusting for other clinical factors, data showed that risk-group was an independent factor for the OS of patients with LUAD (training set: HR = 2.34, 95% CI [1.57–3.5], *P*  <  0.001; validation set: HR = 2.1, 95% CI [1.15–3.9], P = 0.017).

**Table 3:** Univariate Cox regression analysis for TCGA cohort. T stage, N stage, Stage and Risk group were significantly associated with OS.

**Variables**		**Number** **(percent)**	**HR(95CI)**	***P*-Value**
**Age**				0.370
	<65y	136 (42.9%)	reference	
	≥65y	181 (57.1%)	1.18 (0.82–1.70)	0.372
**Gender**				0.455
	Female	163 (51.4%)	reference	
	Male	154 (48.6%)	1.14 (0.80–1.62)	0.455
**Tstage**				*<0.001*
	T1	98 (30.9%)	reference	
	T2	180 (56.8%)	2.0 (1.23–3.23)	0.005
	T3+4	39 (12.3%)	4.02 (2.23–7.23)	<0.001
**Nstage**				*<0.001*
	N0	203 (64.0%)	reference	
	N1	67 (21.1%)	2.028 (1.33–3.09)	<0.001
	N2	47 (14.8%)	3.342 (2.16–5.18)	<0.001
**Stage**				*<0.001*
	I	172 (54.3%)	reference	
	II	86 (27.1%)	1.77 (1.16–2.70)	<0.001
	III	59 (18.6%)	3.44 (2.25–5.28)	<0.001
**Risk_group**				*<0.001*
	Low-risk	159 (50.2%)	reference	
	High-risk	158 (49.8%)	2.727 (1.87–3.98)	<0.001

**Table 4:** Univariate Cox regression analysis for the GSE50081 cohort. T stage, N stage , Stage and Risk group were significantly associated with OS.

**Variables**		**Number** **(percent)**	**HR (95 CI)**	***P*-Value**
**Age**				0.413
	<70y	61 (48.0%)	reference	
	≥70y	66 (52.0%)	1.26 (0.72–2.20)	0.415
**Gender**				0.226
	Female	62 (48.8%)	reference	
	Male	65 (51.2%)	1.41 (0.81–2.46)	0.228
**Tstage**				*<0.001*
	T1	43 (33.9%)	reference	
	T2	82 (64.6%)	2.44 (1.22–4.90)	0.010
	T3+4	2 (1.6%)	11.7 (2.50–55.0)	0.002
**N stage**				*0.008*
	N0	94(74.0%)	reference	
	N1	33 (26.0%)	2.14 (1.20–3.83)	0.010
**Stage**				*0.001*
	I	92 (72.4%)	reference	
	II	35 (27.6%)	2.44 (1.38–4.32)	0.002
**Risk_group**				*<0.001*
	Low-risk	64 (50.4%)	reference	
	High-risk	63 (49.6%)	2.72 (1.52–4.86)	<0.001

### The relationship between risk-group and clinicopathological factors

Table 5 shows the proportions of patients in each risk-group by age, gender, T-stage, N-stage and Stage. It can be found that the high-risk proportion was significantly larger associated with a higher T-stage, N-stage, and Stage. This positive correlation also demonstrated that the risk score increased with tumor progression.

**Table 5:** The relation between risk-group and clinical parameter. The high-risk proportion was significantly larger associated with a higher T-stage, N-stage, and Stage.

**Variables**		**TCGA** **Low risk**	**TCGA** **High risk**	***P*-value**		**GSE50081** **Low risk**	**GSE50081** **High risk**	***P*-value**
**All**		159	158			64	63	
**Age**				0.948				0.932
	<65y	69 (50.7%)	67 (49.3%)		<70y	30 (49.2%)	31 (50.8%)	
	≥65y	90 (49.7%)	91 (50.3%)		≥70y	34 (51.5%)	32 (48.5%)	
**Gender**				*0.049*				1.000
	male	68 (44.2%)	86 (55.8%)		male	33 (50.8%)	32 (49.2%)	
	female	91 (55.8%)	72 (44.2%)		female	31 (50.0%)	31 (50.0%)	
**T-stage**				*<0.001*				*0.004*
	T1	66 (67.3%)	32 (32.7%)		T1	30 (69.8%)	13 (30.2%)	
	T2	81 (45.0%)	99 (55.0%)		T2	34 (41.5%)	48 (58.5%)	
	T3+4	12 (30.8%)	27 (69.2%)		T3+4	0	2 (100%)	
**N-stage**				*0.002*				*0.013*
	N0	116 (57.1%)	87 (42.9%)		N0	54 (57.4%)	40 (42.6%)	
	N1	28 (41.8%)	39 (58.2%)		N1	10 (30.3%)	23 (69.7%)	
	N2	15 (31.9%)	32 (68.1%)					
**Stage**				*0.001*				*0.005*
	I	102 (59.3%)	70 (40.7%)		I	54 (58.7%)	38 (41.3%)	
	II	37 (43.0%)	49 (57.0%)		II	10 (28.6%)	25 (71.4%)	
	III	20 (33.9%)	39 (66.1%)					

### The immune microenvironment differed between risk groups

**Figure 9:**
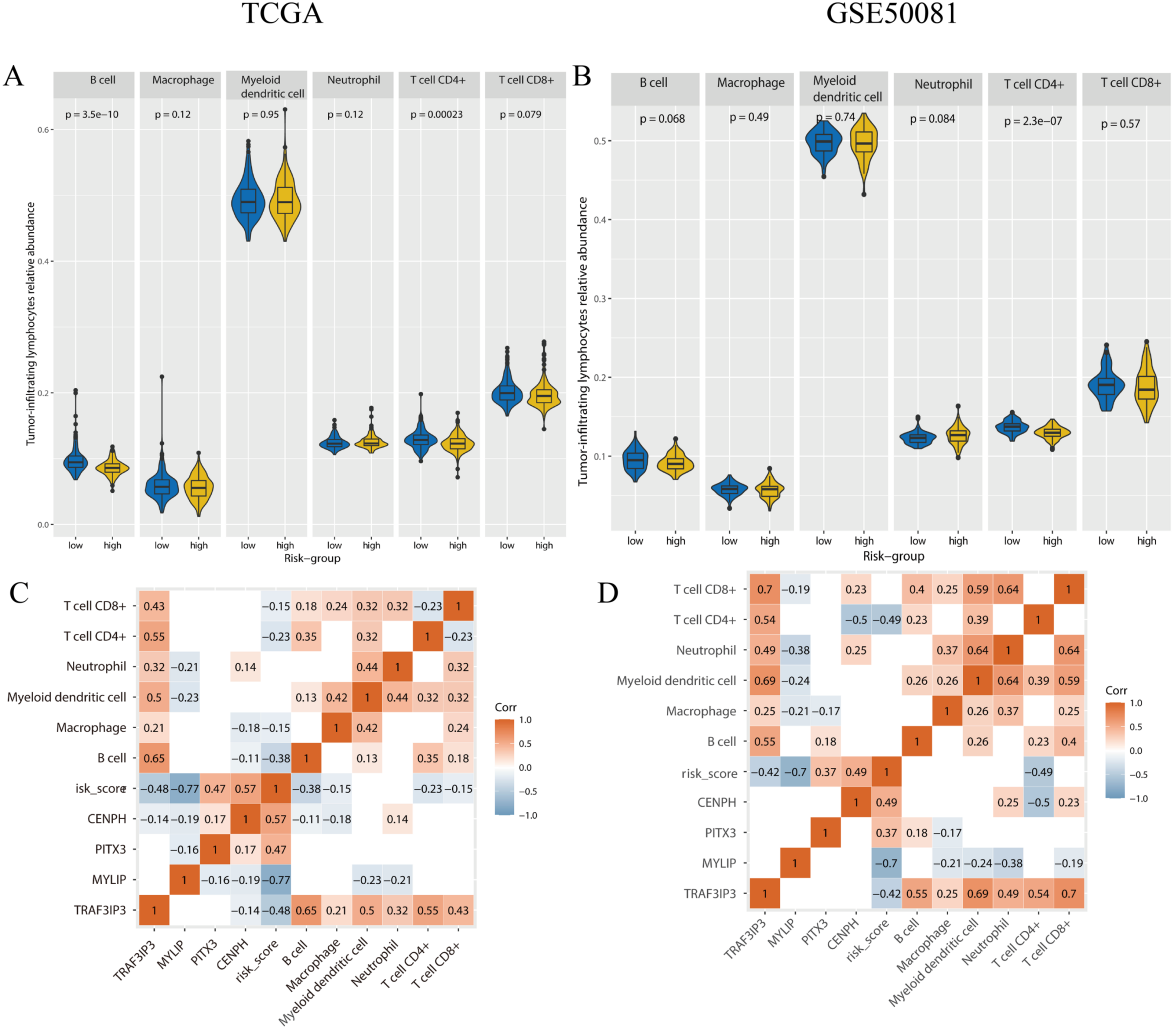
The tumor-infiltrating lymphocytes evaluation and the correlation analysis with genes expression and risk-score. (A, B) The tumor-infiltrating lymphocytes relative abundance for two cohorts using TIMER 2.0. (C, D) The correlation between the tumor-infiltrating lymphocytes relative abundance, gene expression, and risk-score, only the *p*-value < 0.05 were showed. A and C were the TCGA cohort, B and D were the GSE50081 cohort.

To determine if there were any differences with regards to tumor-infiltrating lymphocytes (TILs) relative abundance between high and low risk groups, we applied the online tool TIMER2.0 using gene expression data (Fig. 9). In the training set, the content of B-cells (*p* < 0.001), CD4+ T-cells (*p* < 0.001) was significantly lower in the high-risk group. In the validation set, the number of CD4+ T cells (*p* < 0.001) was significantly lower in the high-risk group.

We performed correlation analysis for the expression levels of the four genes, risk scores, and the relative abundance of TILs, in the two cohorts (Fig. 9). In the training set, the relative abundance of B-cells exhibited a significantly negative correlation with risk score (correlation coefficient = −0.38, *p* < 0.05). There was a negative correlation between CD4+T cells and risk score in both the training set and the validation set (correlation coefficient: −0.23, −0.49, *p* < 0.05). The expression levels of *TAF3IP3* and all tumor-infiltrating lymphocytes were positively correlated in both datasets (correlation coefficient: 0.21–0.7, *p* < 0.05).

### Development and validation of a composite prognostic model

**Figure 10:**
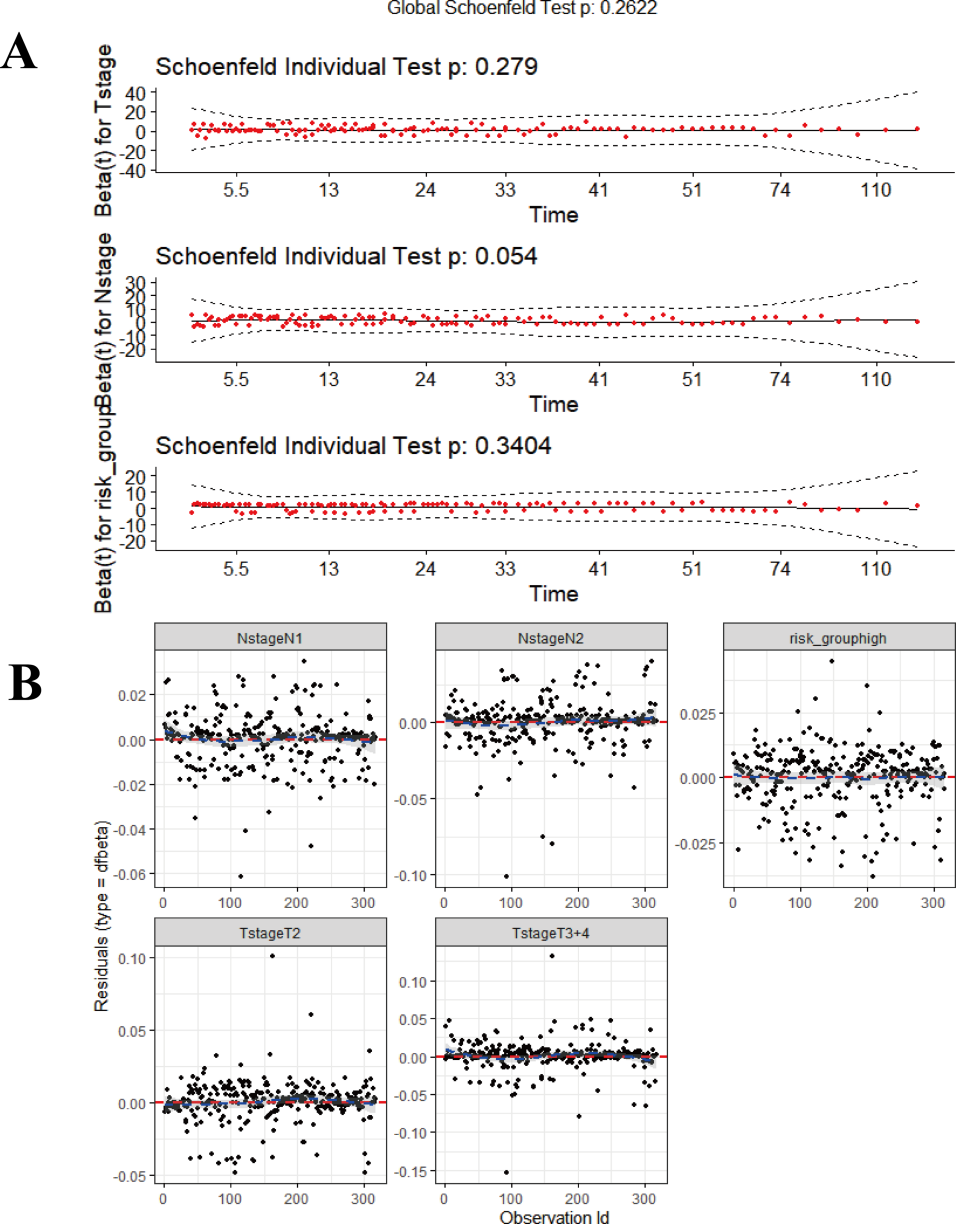
The diagnosis for combined model (M5). (A) Proportional hazards hypothesis test. The curves are smooth, *p*-values of each covariate and global test are all > 0.05, all the covariates satisfies proportional hazards risk hypothesis. (B) The strong impact point test shows a uniform distribution of dfbeta values; none of the outliers affects the model estimates. The ‘survmier’ package was used for this figure.

Data derived from the training were used to develop the final prognostic model. As described previously, T-stage, N-stage, Stage and risk-group were shown to be prognostic factors *via* stepwise regression. We chose T-stage, N-stage and risk-group as parameters to establish the final model and referred to this final model as the composite prognostic model (named M5) which featured a combination of genetic and clinical factors. In addition, we performed model diagnostics for M5. Figure 10 shows that each covariate satisfies the proportional hazards risk hypothesis. None of the outliers affected the model estimates.

**Figure 11:**
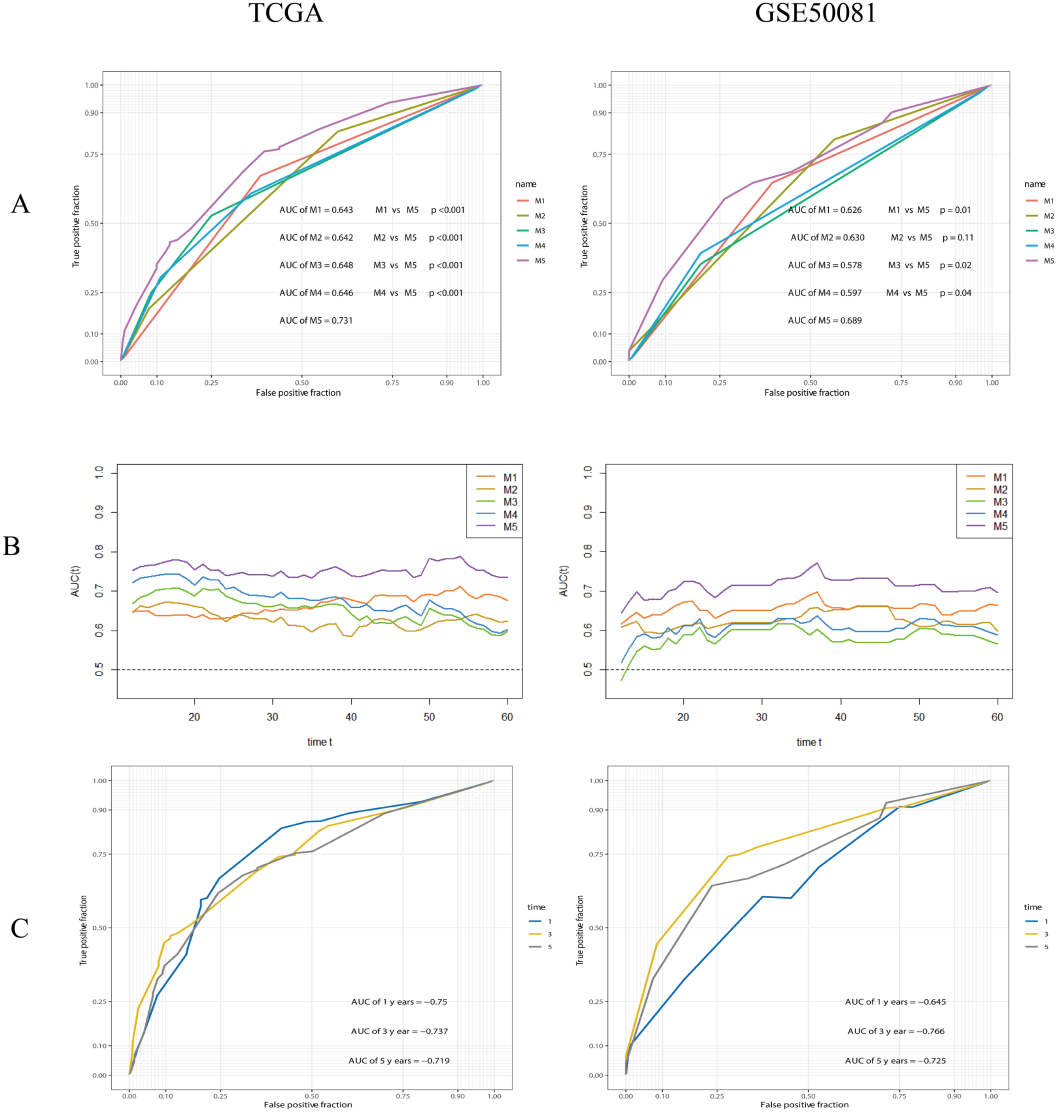
ROC analysis and comparision of models. (A) ROC analysis of models. M1: risk-group; M2: T-stage; M3: N-stage; M4: Stage (TNM stage); M5: the composite prognostic model. The performance of the composite model (M5) was significantly better than others. (B) Time-dependent AUC curves. (C) 1-, 3-, 5-years ROC curves of the composite prognostic model.

The performance of the composite model (AUC-ROC = 0.731) was significantly better than other parameters, including risk-group (M1, AUC-ROC = 0.643, *p* < 0.001), T-stage (M2, AUC-ROC = 0.642, *p* < 0.001), N-stage (M3, AUC-ROC = 0.648, *p* < 0.001), and Stage (M4, AUC-ROC = 0.646, *p* < 0.001). In the validation cohort, the AUC-ROC for M5 was 0.689, higher than other covariates; however, the difference between M2 and M5 was not significant (*p* = 0.11). The AUC values for M5 at 1, 3 and 5 years were 0.750, 0.737 and 0.719, in the training set, and 0.645, 0.766, and 0.725, in the validation set, respectively. Further details are given in Fig. 11.

**Figure 12:**
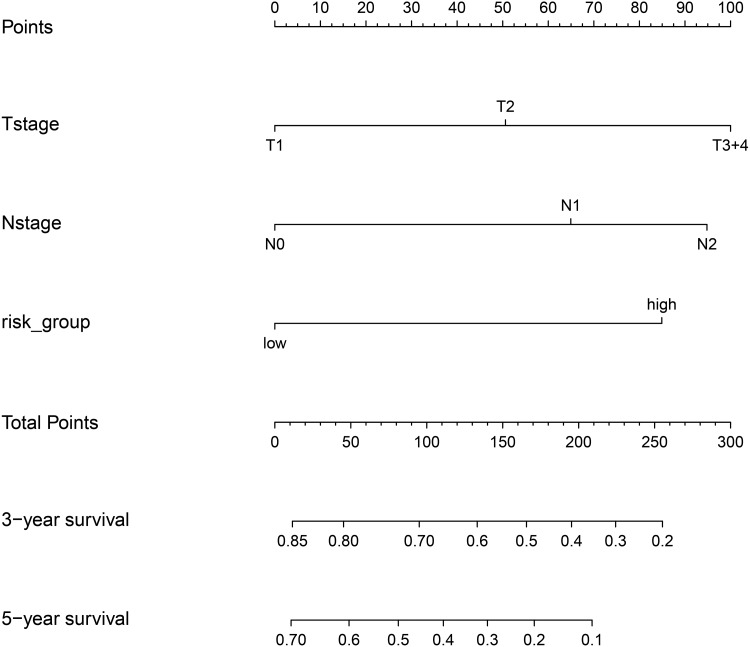
Nomgram plot for the composite prognostic model (M5). According to the total points of Tstage, Nstage and risk_group of each case, the probability of 3-year survival and the probability of 5-year survival can be predicted.

**Figure 13:**
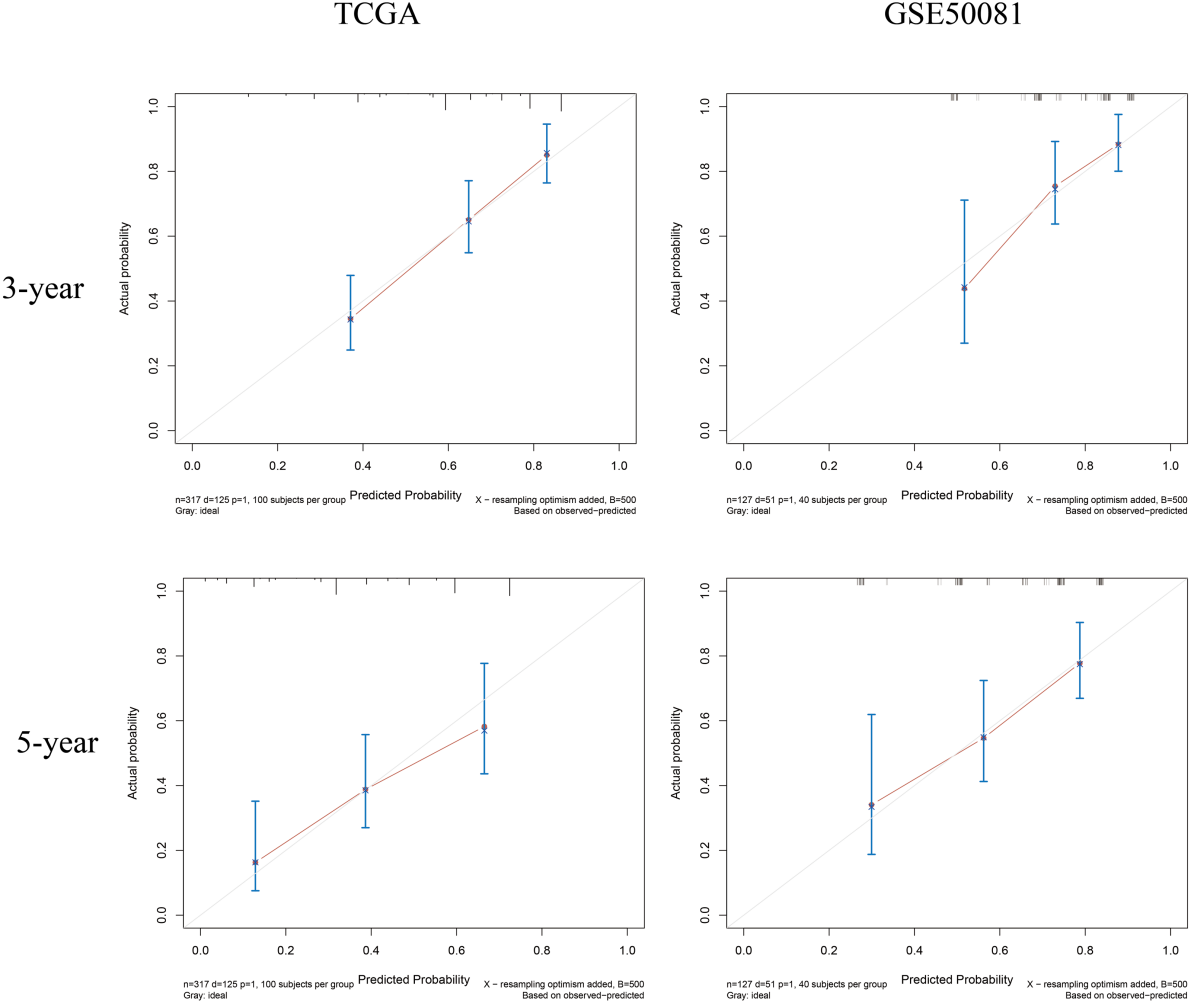
Calibration curves for the composite prognostic model at 3-year and 5-year. The predicted probabilities and the actual probabilities match well.

Finally, we plotted a nomogram and calibration curves for the composite prognostic model (Fig. 12). The calibration curves showed a good match between the predicted probabilities and the actual probabilities (Fig. 13).

## Discussion

Several studies have reported the predictive value of gene expression for the prognosis of patients suffering from cancer, including breast, colorectal, lung, gastric, and ovarian cancer (Li et al., 2020a; Pei et al., 2020; Xie et al., 2020; Zhang et al., 2020b). Most of these studies involved the selection of differentially expressed genes followed by the screening of prognosis-related genes. However, this practice may have omitted some genes that are not differentially expressed but are important for prognosis. In the present study, we performed prognostic gene screening directly from gene expression profiles. Using a random grouping method, only 17 genes were initially identified as stable prognostic genes; finally, we identified four genes (*CENPH*, *MYLIP*, *PITX3*, and *TRAF3IP3)* that could be used as a reliable signature. Only the expression levels of the *CENPH* gene were found to be significantly different when compared between lung adenocarcinoma and normal tissue. Multivariate Cox regression analysis suggested that the four-gene signature represents a reliable and independent tool for predicting prognosis. Subgroup analysis showed that this signature could represent an index for screening out high-risk samples from different Stages and provide new ideas for treatment decisions.

Next, we compared the prognostic ability of risk-group and other parameters. The TNM system (M4 in the present study) is the most widely used cancer staging system and is commonly used to evaluate prognosis and facilitate clinical treatment decisions. In the present study, the high-risk group was more often associated with a high T-stage, N-stage, and Stage, thus indicating that there is a relationship between risk score and TNM stage. We analyzed the discriminatory ability of risk-group (M1), T-stage (M2), N-stage (M3), Stage (M4), and a combined model (M5). In the training set, M5 had a better discriminatory ability than others and exhibited the best performance across all time points. In the validation set, M5 was significantly better than other indicators except for M2, which did not reach a significant difference compared to M5, but was close to a significant difference (*p* = 0.11). However, analysis showed that the combined model including the gene signature was more reliable than the traditional TNM system (M4) at any time point.

TILs are correlated with the prognosis of several cancer. A recent meta-analysis demonstrated that high-density TILs, CD3+ TILs, CD4+ TILs, CD8+ TILs and CD20+ TILs in cancer nests were good prognostic markers for NSCLC patients (Chen et al., 2020). Our current analysis showed that the high-risk group featured a significantly lower abundance of CD4+ T cells, both in the training and validation groups. The abundance of B-cells and CD8+ T cells also tended to be lower in the high-risk group; however, only the difference in B-cells reached statistical significance in the training set. This suggests the existence of a correlation between risk-scores and the tumor immune microenvironment, particularly with CD4+ T cells.

We identified that *TRAF3IP3* was positively correlated with all six types of immune infiltrating lymphocytes, thus suggesting that this gene is closely related to tumor immune infiltration. However, very few studies in the existing literature have investigated the role of *TRAF3IP3*. One previous study reported that TRAF3 interacting protein 3 (TRAF3IP3) is expressed in the immune system and known to be involved in cell maturation, tissue development, and immune response (Nasarre et al., 2018). TRAF3IP3 is known to regulate the development of thymocytes *via* the ERK signaling pathway (Zou et al., 2015). The establishment of TRAF3IP3-knockout mice revealed a significant reduction in the number of common lymphoid progenitor cells in the bone marrow; furthermore, the there was a total absence of B cells in the marginal zone of the spleen (Peng et al., 2015). We also showed that tumor-infiltrating B cells were also positively correlated with *TRAF3IP3* expression; B cells have also been reported to be actively proliferating in tumors, This suggests that the regulatory system for this gene in B cells may also be present in the tumor microenvironment. Furthermore, our research showed that the extent of tumor-infiltrating CD4+ T cells was positively correlated with the expression of *TRAF3IP3* (correlation coefficients of 0.55 and 0.54 for the training and validation sets, respectively). We speculate that the effect of *TRAF3IP3* may be related to its positive regulation of tumor-infiltrating immune cells.

The expression of *CENPH* is known to be elevated and associated with the progression of many cancers, such as oral, tongue, nasopharyngeal, hypopharyngeal, lung, breast, esophageal, gastric, colorectal, hepatocellular, and renal cancer (Wu et al., 2015). Chinese researchers were the first to report the relationship between *CENPH* and NSCLC and showed that both mRNA levels and protein levels were over-expressed in cases of lung cancer. Further analysis showed that high expression levels of CENPH protein were positively correlated with Ki-67 and associated with a poor prognosis, particularly in patients diagnosed with stage I–II lung cancer (Liao et al., 2009). Our analysis also showed that *CENPH* was overexpressed in cases of lung adenocarcinoma and correlated with a poor prognosis. Furthermore, the expression levels of *CENPH* gradually increased in stages I–IV, suggesting that the expression of this gene is closely related to tumor progression. The *MYLIP* gene, also known as *IDOL* or *MIR*, encodes a protein that belongs to the cluster of cytoskeletal proteins and is known to play a role in regulating the motility, migration and adhesion of cells; the inhibition of *MYLIP* expression has also been shown to promote migration and metastasis of breast and cervical cancer cells (Ni et al., 2020; Zhao et al., 2017). *MYLIP* represents a potential marker and target for the diagnosis and treatment of breast cancer (Zhao et al., 2020). In our analysis, the expression levels of *MYLIP* were significantly higher in the lymph node negative group than in the lymph node positive group (*p* = 0.0052 and 0.0087 for the training and validation sets, respectively) and therefore represented a prognostic protective gene. This finding suggested that high levels of *MYLIP* expression may reduce the migratory ability of lung adenocarcinoma cells; this finding was consistent with previous studies. *PITX3* is a member of the PITX gene family, which includes *PITX1*, *PITX2*, and *PITX3*, all of which are related to lung cancer (Tran & Kioussi, 2021; Zhang et al., 2021). There are few studies on the occurrence and development of *PITX3* and lung cancer. However, previous studies have shown that the mRNA expression level of *PITX3* in lung cancer tissue is not significantly different from normal lung tissue, but it is closely related to tumor stage, and the prognosis of patients with high expression is poor (Zhang et al., 2021). In this study, it is also shown that *PITX3* is a poor prognostic gene, but the specific mechanism is still unclear. In summary, the gene sets we identified were associated with immune infiltration along with the proliferation and migration of lung adenocarcinoma.

Although we identified four genes associated with the prognosis of patients with LUAD using bioinformatics technology and large sample sizes, our research still has some limitations that need to be considered. First, the platforms applied in the TCGA and GEO cohorts, and the reported gene expression levels for these cohorts, were different. This led to large differences in risk scores between the two sets, thus forcing us to use different cutoff values when risk grouping. This may limit the external application of our model. Second, three of the four genes we identified were down-regulated in tumors when compared to normal tissues but with no statistical difference; consequently, it may be difficult to perform immunohistochemical validations in future. Third, conclusions derived from our bioinformatic analyses were not validated by our cohort trials. Thus, our model needs to be extrapolated further in larger studies.

## Conclusions

In conclusion, we developed a risk-score model featuring a four gene signature that was related to immune function, cell proliferation, and cell migration. This risk-score represented an independent prognostic factor in both the training and validation sets. In terms of assessing prognosis, we should also pay attention to genes that are related to survival, even if their expression levels do not differ from normal tissues. On this basis, clinical factors were combined to construct a new combined prognostic prediction model with good discrimination and calibration; this combined model performed well in the validation set. This combined model may be important for predicting the prognosis of patients with lung adenocarcinoma.

##  Supplemental Information

10.7717/peerj.11911/supp-1 Supplemental Information 1 Raw data of the TCGA cohort for correlation analysis between genetic signature and clinical factors The raw data type of gene expression RNAseq downloaded from the public database was FPKM, while the gene expression data in this table was standardized, using z-score function. All the samples were primary tumor.

10.7717/peerj.11911/supp-2 Supplemental Information 2 Raw data of the GSE50081 cohort for correlation analysis between genetic signature and clinical factors The raw data type of gene expression RNAseq downloaded from the public database was FPKM, while the gene expression data in this table was standardized, using z-score function. All the samples were primary tumor.

## Additional Information and Declarations

### Data Availability

The following information was supplied regarding data availability: The data are available at NCBI GEO: GSE50081 and from UCSC Xena:
https://xenabrowser.net/datapages/?dataset=TCGA-LUAD.htseq_fpkm.tsvhost=https%3A%2F%2Fgdc.xenahubs.netremoveHub=https%3A%2F%2Fxena.treehouse.gi.ucsc.edu%3A443

https://xenabrowser.net/datapages/?dataset=TCGA-LUAD.GDC_phenotype.tsvhost=https%3A%2F%2Fgdc.xenahubs.netremoveHub=https%3A%2F%2Fxena.treehouse.gi.ucsc.edu%3A443
https://xenabrowser.net/datapages/?dataset=TCGA-LUAD.survival.tsvhost=https%3A%2F%2Fgdc.xenahubs.netremoveHub=https%3A%2F%2Fxena.treehouse.gi.ucsc.edu%3A443.
